# Low-dose aspirin and rivaroxaban combination therapy to overcome aspirin non-sensitivity in patients with vascular disease

**DOI:** 10.3389/fcvm.2022.912114

**Published:** 2022-08-11

**Authors:** Hamzah Khan, Mariya Popkov, Shubha Jain, Niousha Djahanpour, Muzammil H. Syed, Margaret L. Rand, John Eikelboom, C. David Mazer, Mohammed Al-Omran, Rawand Abdin, Mohammad Qadura

**Affiliations:** ^1^Division of Vascular Surgery, St. Michael's Hospital, Toronto, ON, Canada; ^2^Department of Laboratory Medicine and Pathobiology, Biochemistry and Pediatrics, University of Toronto, Toronto, ON, Canada; ^3^Division of Haematology/Oncology, Translational Medicine, Research Institute, The Hospital for Sick Children, Toronto, ON, Canada; ^4^Department of Medicine, McMaster University, Hamilton, ON, Canada; ^5^Population Health Research Institute, McMaster University, Hamilton, ON, Canada; ^6^Department of Anesthesia, Li Ka Shing Knowledge Institute, St. Michael's Hospital, Toronto, ON, Canada; ^7^Departments of Anesthesiology, Pain Medicine and Physiology, University of Toronto, Toronto, ON, Canada; ^8^Keenan Research Centre for Biomedical Science, Li Ka Shing Knowledge Institute of St. Michael's Hospital, Toronto, ON, Canada; ^9^Department of Surgery, King Faisal Specialist Hospital and Research Center, Riyadh, Saudi Arabia

**Keywords:** rivaroxaban, aspirin, antiplatelet, non-sensitivity, vascular

## Abstract

Approximately 20% of vascular patients treated with acetyl salicylic acid (i.e., aspirin) demonstrate less than expected platelet inhibition – putting them at a four-fold increased risk of adverse cardiovascular events. Low-dose rivaroxaban (2.5 mg twice daily) in combination with low-dose aspirin has been shown to reduce adverse cardiovascular and limb events when compared to aspirin alone. In this study, light transmission aggregometry was used to measure arachidonic acid-induced platelet aggregation to evaluate the potential of combining low-dose rivaroxaban and aspirin in attenuating or overcoming aspirin non-sensitivity. In the discovery phase, 83 patients with peripheral arterial disease (PAD) taking 81 mg aspirin daily were recruited from the outpatient vascular surgery clinic at St Michael's Hospital between January to September 2021. 19 (23%) were determined to be non-sensitive to aspirin. After *ex-vivo* addition of 2.5 mg dosage equivalent of rivaroxaban, aspirin non-sensitivity was overcome in 11 (58%) of these 19 patients. In the validation phase, 58 patients with cardiovascular risk factors who were not previously prescribed aspirin were recruited. In this group, *ex-vivo* addition of 2.5 mg dosage equivalent of rivaroxaban significantly reduced arachidonic acid-induced platelet aggregation in the presence of aspirin. These results demonstrate the potential for low-dose rivaroxaban to overcome aspirin non-sensitivity in patients with PAD. Further studies are needed to evaluate and confirm these findings.

## Introduction

Aspirin non-sensitivity affects 1 in 4 patients with cardiovascular disease (1–4). It is defined as lower than expected platelet inhibition by aspirin on laboratory testing that results in an increased risk of cardiovascular events (5, 6). Aspirin non-sensitive patients are at a higher risk of stroke, myocardial infarction and death (7, 8). Despite the scope of the issue, there are no proven treatments to overcome aspirin non-sensitivity.

Rivaroxaban is effective for the prevention and treatment of venous thromboembolism, prevention of stroke in atrial fibrillation, and the prevention of atherothrombotic events in patients with coronary artery disease (CAD) or peripheral arterial disease (PAD) (9, 10). In patients with PAD, the combination therapy of aspirin and rivaroxaban reduced major adverse cardiovascular events (MACE - stroke, myocardial infarction, and cardiovascular related death) by 28% and major adverse limb events (MALE- chronic limb threatening ischemia, arterial bypass and amputation) by 46% (10). However, the potential for this combination therapy to overcome aspirin non-sensitivity is yet to be investigated.

In this pilot study, we explored the potential for low-dose rivaroxaban to overcome aspirin non-sensitivity *ex vivo* in patients with PAD.

## Materials and methods

### Ethics approval

This study was performed in accordance with the Declaration of Helsinki and was approved by the Unity Health Toronto Research Ethics Board at St. Michael's Hospital, Toronto, Canada (REB #19-241, September 4, 2020). All patients provided written informed consent before participating in this study.

### *Ex-vivo* assessment of rivaroxaban's ability to overcome aspirin non-sensitivity

#### Selection of patients with peripheral arterial disease taking aspirin (discovery phase)

In the discovery phase, consecutive patients with PAD on 81 mg aspirin daily presenting to ambulatory vascular surgery clinics at St. Michael's Hospital between January to September 2021 were recruited. Patients' past medical history and clinical characteristics were recorded. Diagnosis of PAD was established clinically (through history and physical exam) or through utilizing the ankle brachial index (ABI) <0.9. Patients were deemed ineligible if they met one or more of the following criteria: patients taking any antithrombotic medication other than 81 mg aspirin daily; patients with history of bleeding disorders, gastrointestinal bleeding, thrombocytopenia, anemia, or leukopenia; patients who were pregnant, or patients under the age of 18.

#### Baseline data collection

Each patient was interviewed to obtain a full medical history, including previous history of heart disease, smoking status, hypercholesterolemia, hypertension, renal disease, history of cerebrovascular events, and current medications. As previously described (11), patients with HbA1c ≥6.5% or using anti-diabetic medication were considered as having diabetes mellitus. Patients with total cholesterol >5.2 mmol/L or triglyceride >1.7 mmol/L, or using antihyperlipidemic medication were considered as having hypercholesterolemia. Patients using antihypertensive medication or with a systolic blood pressure ≥130 mmHg or a diastolic pressure ≥80 mm Hg were considered as hypertensive. Patients with an estimated glomerular filtration rate of <60 mL/min/1.73 m^2^ were considered to have renal disease (11).

#### Blood sample collection and light transmission aggregometry

Blood was drawn from an antecubital vein using a 21-gauge needle into 2.7 mL 3.2% sodium citrate tubes from patients within 24 h of aspirin intake. Platelet aggregation testing was started within 15 min of the blood draw. Light transmission aggregometry (LTA) analysis was conducted as per previous protocols (12, 13). In short, citrated-blood was centrifuged at 300xg for 7 min to collect platelet-rich plasma (PRP). A separate tube of autologous citrated whole blood was spun at 1200xg for 10 min to collect platelet-poor plasma (PPP). Platelet counts were adjusted to 2–3 × 10^9^ /mL using autologous PPP (12, 14). LTA was conducted on an AggRam Analyzer (Helena Laboratories, Beaumont TX, USA), set to read PPP as 100% light transmission, and PRP as 0%, with aggregation proportional to light transmission. Platelets were activated with 0.5 mg/mL arachidonic acid (101297, Bio/Data Corporation, Horsham). Testing was conducted at 37°C with the stir rate set to 1,000 rpm. Baseline light transmission was determined for 30 s, after which arachidonic acid was added and aggregation recorded for 10 min. Samples were run in duplicates and mean maximal platelet aggregation was recorded. Patients were considered aspirin non-sensitive if they had a maximal platelet aggregation of ≥20% when activated with arachidonic acid, as per previous studies (13–17).

#### Aspirin sensitivity testing after *ex-vivo* addition of rivaroxaban

Sensitivity to aspirin after incubation with 2.5 mg rivaroxaban was determined using LTA. Non-sensitive patients' autologous PRP samples were spiked with a final concentration of 50 ug/mL of rivaroxaban, which equates to maximum plasma concentration (cmax) after ingestion of 2.5 mg rivaroxaban as previously described by Mueck et al. (18) and Kreutz et al. (19), and the samples were incubated at 37°C for 15 min before testing. As mentioned above, platelet aggregation was tested with LTA, and maximal aggregation and aspirin sensitivity were determined. Our protocol was optimized to account for the effects of the buffer on platelet aggregation.

### *Ex-vivo* assessment of the additive effects of rivaroxaban and aspirin

#### Selection of patients with vascular risk factors not taking aspirin (validation phase)

For the validation phase, we assessed the effects of 2.5 mg rivaroxaban alone on platelet function. A new patient cohort with cardiovascular risk factors not taking aspirin was recruited. Patients were deemed ineligible if they met one or more of the following criteria: patients taking any antiplatelet drug or anticoagulant medication; patients with history of bleeding disorders, gastrointestinal bleeding, thrombocytopenia, anemia, or leukopenia; patients who were pregnant, or patients under the age of 18.

#### Blood sample collection and light transmission aggregometry

**Figure 1:**
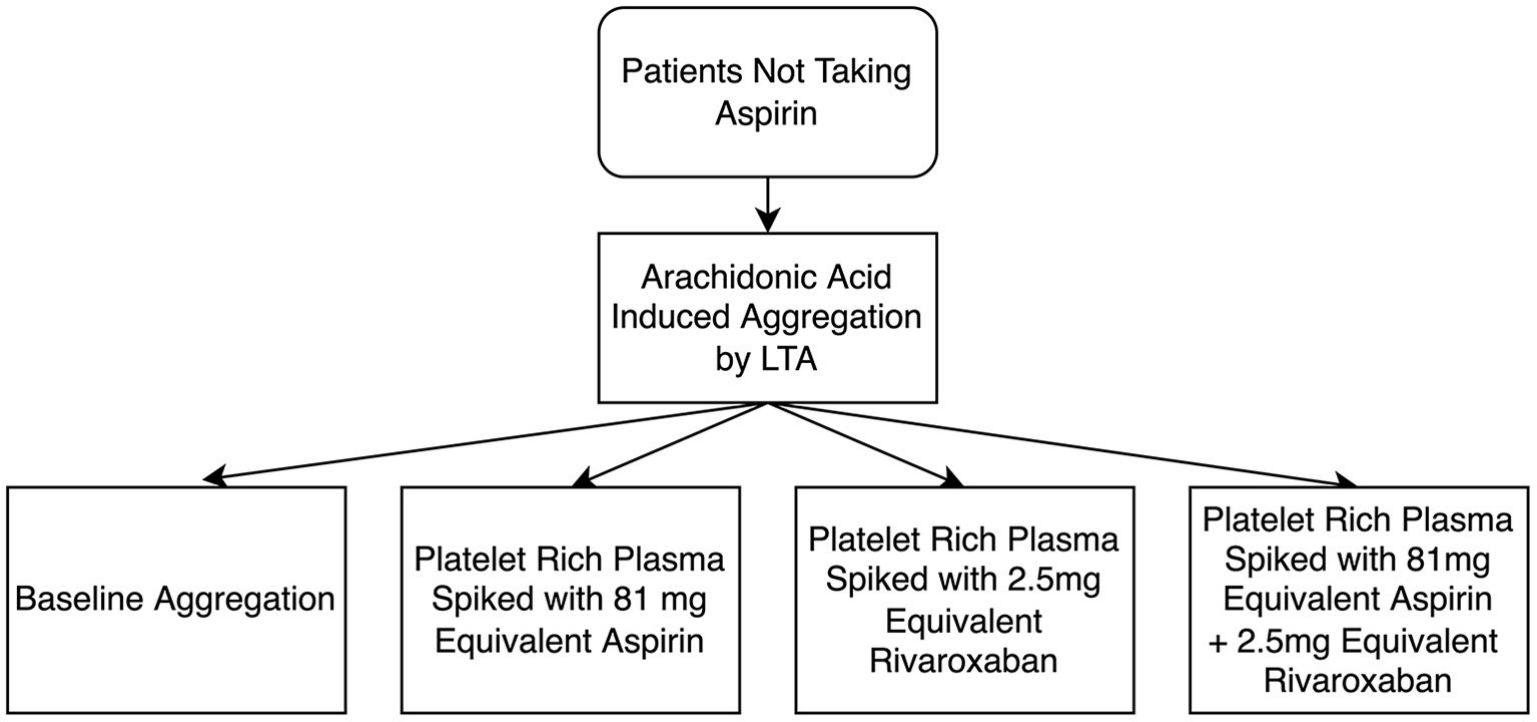
Flow diagram of light transmission aggregometry (LTA) testing of the antiplatelet effects of rivaroxaban, and the additive effects between rivaroxaban and aspirin. A final concentration of 50 μg/mL was used as 2.5 mg equivalent rivaroxaban and a final concentration of 10 μM was used as 81 mg equivalent aspirin.

Next, PRP was collected and arachidonic acid-induced platelet aggregation was measured in 4 different samples: (1) PRP; (2) PRP spiked with 2.5 μL of a rivaroxaban solution to give a final concentration of 50 ug/mL (2.5 mg equivalent) rivaroxaban 3) PRP spiked with 2.5 μL of an aspirin solution to give a final concentration of 10 μM (81 mg equivalent) aspirin; and 4) PRP spiked with both 2.5 mg equivalent rivaroxaban, and 81 mg equivalent aspirin (refer to Figure 1). After spiking, PRP samples were incubated at 37°C for 15 min before testing. LTA was conducted as described above. Patients were considered aspirin non-sensitive if they had a maximal platelet aggregation of ≥20% when activated with arachidonic acid, as per previous studies (8, 13–17, 20, 21).

### Statistical analysis

All clinical characteristics and demographics were presented as frequency and percentages, or mean and standard deviations. Normality was assessed using normality plots and the Shapiro–Wilk test. Normally distributed continuous variables were presented as mean and standard deviation. Non-normally distributed data were presented as median and interquartile range (IQR). Categorical variables were presented as count and percent. The non-parametric Wilcoxon rank sum test was used to compare baseline aggregation with aggregations spike with aspirin and/or rivaroxaban. All hypothesis testing was carried out at the 5% (2-sided) significance level. Statistical analyses were conducted using GraphPad Prism, version 8.4.2.

## Results

### *Ex-vivo* assessment of rivaroxaban's ability to overcome aspirin non-sensitivity

#### Baseline characteristics of the discovery phase cohort

In the discovery phase, 83 patients with PAD prescribed 81 mg aspirin daily were recruited. As shown in Table 1, the patient group had a mean age of 67 years, primarily consisted of males (63%), and had high rates of cardiovascular risk factors such as hypertension (75%), hypercholesterolemia (75%), diabetes mellitus (33%), and smoking (80%).

**Table 1:** Clinical characteristics and demographics of patients taking 81 mg aspirin.

**Variable**	**Patients taking 81 mg aspirin daily (*n* = 83)**
Age (yrs)	67 ± 12
Sex (*n*, % Male)	52 (63)
Hypertension (*n*, %)	62 (75)
Hypercholesterolemia (*n*, %)	62 (75)
Diabetes (*n*, %)	27 (33)
Smoking Hx (*n*, %)	66 (80)
CAD (*n*, %)	18 (22)
Stroke/TIA (*n*, %)	8 (10)
Statin (*n*, %)	62 (76)
ACEi/ARB (*n*, %)	46 (55)
B-blocker (*n*, %)	24 (29)

*History (Hx); Transient Ischemic Attack (TIA); Coronary artery disease (CAD); angiotensin-converting enzyme (ACE) inhibitors (ACEi/ARB); Beta blockers (B-blocker). Age is presented as mean ± standard deviation and categorical variables are presented as counts and percentages*.

#### Aspirin sensitivity testing

Of the 83 patients, 19 patients (23%) had a maximal platelet aggregation ≥20% in response to arachidonic acid, despite taking 81 mg aspirin with a mean maximal platelet aggregation of 42 (95% CI; 30, 56). These 19 patients were considered aspirin non-sensitive. Non-sensitive patients were more likely to be taking an angiotensin converting enzyme inhibitor/angiotensin II receptor blocker (ACEi/ARB) when compared to sensitive patients. There was no significant difference in any of the other characteristics measured (Table 2).

**Table 2:** Comparison of clinical characteristics and demographics between patients sensitive and non-sensitive to 81 mg aspirin.

**Variable**	**Patients sensitive to 81 mg aspirin (*n* = 64)**	**Patients non-sensitive to 81 mg Aspirin (*n* = 19)**	***p*-value**
Age (yrs)	68 ± 12	66 ± 11	0.545
Sex (*n*, % Male)	38 (59)	14 (74)	0.294
Hypertension (*n*, %)	49 (77)	13 (68)	0.551
Hypercholesterolemia (*n*, %)	49 (77)	13 (68)	0.551
Diabetes (*n*, %)	20 (31)	7 (37)	0.099
Smoking Hx (*n*, %)	51 (80)	15 (79)	>0.999
CAD (*n*, %)	16 (25)	2 (11)	0.221
Stroke/TIA (*n*, %)	5 (8)	3 (16)	0.376
Statin (*n*, %)	50 (78)	13 (68)	0.252
ACEi/ARB (*n*, %)	40 (63)	6 (32)*	0.021
B-blocker (*n*, %)	20 (31)	4 (21)	0.566

Coronary artery disease (CAD); angiotensin-converting enzyme (ACE) inhibitors (ACEi/Arb); Beta blocker (B-blocker). Continuous variables are presented as mean ± standard deviation and categorical variables are presented as counts and percentages. ^*^Represents a significant difference between aspirin sensitive and non-sensitive patients with p < 0.05.

#### *Ex-vivo* effects of rivaroxaban on platelet function in aspirin non-sensitive patient

**Figure 2:**
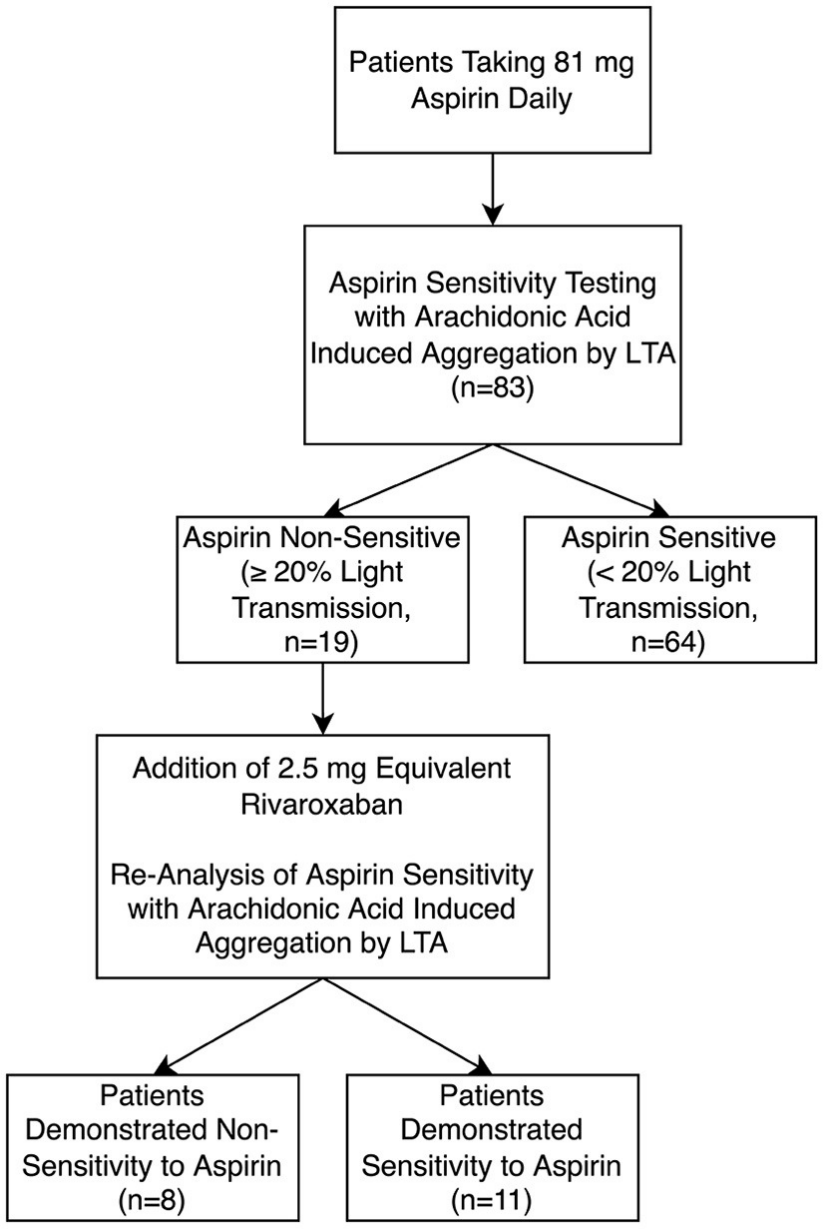
Aspirin sensitivity before and after incubation with low-dose 2.5 mg equivalent rivaroxaban (*n* = 83) using light transmission aggregometry (LTA).

In this experiment, a fresh sample of PRP from the 19 patients non-sensitive to 81 mg aspirin was spiked with low-dose 2.5 mg rivaroxaban dosage equivalent (Figure 2). In the presence of rivaroxaban, 11 (58%) of the previously known 19 aspirin non-sensitive patients demonstrated a maximal platelet aggregation <20%, indicating they were now aspirin sensitive. Only 8 of the 19 patients continued to have maximal platelet aggregation ≥20%, suggesting that they remained as aspirin non-sensitive (Figure 2). Our data suggest that the addition of 2.5 mg of rivaroxaban helps overcome aspirin non-sensitivity in 58% of patients who were originally non-sensitive to aspirin.

### *Ex-vivo* assessment of the combined effects of rivaroxaban and aspirin (validation phase)

#### Investigating the combined effects of aspirin and rivaroxaban

To further investigate the potential of rivaroxaban overcoming aspirin non-sensitivity, an independent cohort of 58 consecutive patients with vascular risk factors not taking aspirin or any other antiplatelet/anticoagulant drug were recruited in the validation phase. Mean age of this group was 56 years, 62% of patients were male, 60% were smokers, 43% having hypertension, 28% having hypercholesterolemia, and 12% having diabetes mellitus (Table 3).

**Table 3:** Clinical characteristics and demographics of patients not taking aspirin (validation group).

**Variable**	**Patients not on aspirin (*n* = 58)**
Age (yrs)	55 ± 20
Sex (*n*, % Male)	36 (62)
Hypertension (*n*, %)	25 (43)
Hypercholesterolemia (*n*, %)	16 (28)
Diabetes (*n*, %)	7 (12)
Smoking Hx (*n*, %)	35 (60)
CAD (*n*, %)	1 (2)
Stroke/TIA (*n*, %)	2 (3)
Statin (*n*, %)	15 (26)
ACEi/ARB (*n*, %)	12 (21)
B-blocker (*n*, %)	6 (10)

*Coronary artery disease (CAD); angiotensin-converting enzyme (ACE) inhibitors (ACEi/Arb); Beta blockers (B-bl). Continuous variables are presented as mean ± standard deviation and categorical variables are presented as counts and percentages*.

#### Platelet aggregation testing

**Figure 3:**
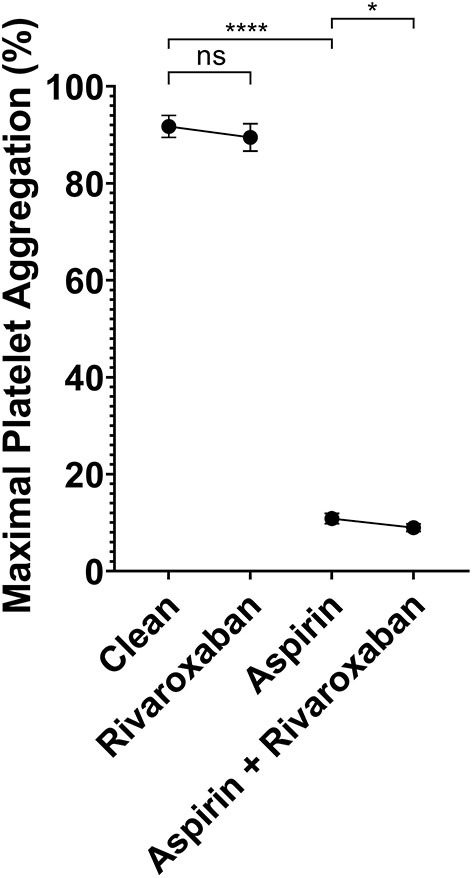
Light transmission aggregometry analysis of platelet inhibition by 2.5 equivalent mg rivaroxaban and/or 81 mg aspirin in autologous platelet rich plasma in response to 0.5 μg/mL arachidonic acid. Maximal light transmission following platelet activation by arachidonic acid in; baseline (control PRP) with no antiplatelet compared to PRP spiked with 2.5 mg equivalent rivaroxaban (50 μg/mL), and PRP spiked with 81 mg equivalent aspirin (ASA - 10 μM) compared to PRP spiked with both 81 mg equivalent aspirin and 2.5mg equivalent rivaroxaban. *p* < 0.05 represented by (*), and *p* < 0.001 represented by (****).

To determine if rivaroxaban has combined effects with aspirin, platelet aggregation in response to arachidonic acid was tested in 4 different PRP samples. First, PRP from patients not taking any antiplatelet or anticoagulant was compared with PRP spiked with 2.5 mg equivalent rivaroxaban. Secondly, PRP was spiked with 81 mg aspirin dosage equivalent, and compared to PRP spiked with both aspirin and rivaroxaban. Compared to non-spiked, control PRP, rivaroxaban alone did not demonstrate a significant reduction in platelet aggregation in response to arachidonic acid (Figure 3). When PRP was spiked with aspirin in the absence or presence of rivaroxaban, rivaroxaban significantly reduced arachidonic acid-induced platelet aggregation compared to aspirin alone. These results show that 2.5 mg rivaroxaban significantly inhibits arachidonic acid-induced platelet aggregation in the presence of aspirin compared to in its absence.

## Discussion

In the discovery phase, we report that approximately 23% of patients with PAD were non-sensitive to their 81 mg aspirin. *Ex-vivo* addition of low-dose 2.5 mg rivaroxaban dosage equivalent successfully overcame aspirin non-sensitivity in 58% of the initial “aspirin non-sensitive” PAD patients. In the validation phase, rivaroxaban was able to significantly reduce platelet aggregation in combination with aspirin. We observed reduction of platelet aggregation in the presence of rivaroxaban alone, but this reduction was not statistically significant.

Large randomized trials have demonstrated that the combination of rivaroxaban (2.5 mg twice daily) and low-dose aspirin (81 mg once daily) compared with aspirin alone reduces major adverse cardiovascular events in patients with coronary artery disease (CAD) or PAD (10, 22, 23). Patients who are non-sensitive to aspirin are at a significantly increased risk of both major adverse limb events and major adverse cardiovascular events, with a hazard ratio of 3.68 (7, 8). Aspirin non-sensitivity can occur in some patients due to epigenetic or phenotypic difference between the COX enzyme and therefore increasing aspirin dose from 81 to 325mg will not be an effective strategy to combat this issue, and hence alternatives such as rivaroxaban, or other antiplatelets would be necessary. Our finding suggests that patients who are non-sensitive to aspirin may derive the greatest benefit from this combination therapy.

Our data allude to combined effects between aspirin and rivaroxaban on platelet aggregation, however several studies have shown other platelet activation pathways that may be influenced by rivaroxaban. For example, Perzborn et al. reported that rivaroxaban inhibited tissue factor (TF)-induced platelet aggregation (24). Subsequently, the authors showed that rivaroxaban inhibited ADP, collagen, and thrombin receptor activating peptide (TRAP)-induced platelet aggregation in a plasma dependent manner, as this inhibition was not seen in the absence of plasma (i.e., washed platelets) (25). The authors concluded that aggregation may be inversely related to dosage, with higher doses associated with reduced platelet aggregation (25). A recent publication by Jurk et al. (26) has also demonstrated that rivaroxaban reduces platelet activation through the PAR-1 receptor, a receptor for thrombin induced platelet activation (26). In our study, we demonstrated that there may be an additional mechanism of platelet inhibition by rivaroxaban through the arachidonic acid pathway, affecting the formation of pro-aggregatory TxA2. The results may also be explained by rivaroxaban's effects on alternative pathways in combination with its effects on the arachodicnic acid pathway causing a combined reduction on platelet aggregation.

This pilot study has some limitations. First, a small sample size was utilized in this study. Further research with larger samples sizes is warranted to confirm our findings. Second, our experiments were limited to *ex-vivo* settings. *In-vivo* analysis of the reduction of platelet activity, and reversal of aspirin non-sensitivity are needed to confirm *ex vivo* findings, as well as to determine if this is linked to a reduction in adverse events in aspirin non-sensitive patients. Lastly, patients were questioned on compliance to daily aspirin therapy, however no testing was conducted to ensure compliance. This may lead to an increase percentage of non-sensitive patients who are actually non-compliant.

In conclusion, we were able to demonstrate that low-dose, 2.5 mg equivalent rivaroxaban was able to significantly reduce arachidonic acid-induced platelet aggregation in the presence of aspirin *ex-vivo* and overcome aspirin non-sensitivity in 58% of patients previously non-sensitive to aspirin. The addition of 2.5 mg rivaroxaban twice daily, in addition to 81 mg aspirin daily, may help to reduce the increased risk of cardiovascular events in patients non-sensitive to aspirin and further large studies are needed to confirm our findings in a clinical setting.

## Data availability statement

The raw data supporting the conclusions of this article will be made available by the authors, without undue reservation.

## Ethics statement

The studies involving human participants were reviewed and approved by Unity Health Toronto Research Ethics Board at St. Michael's Hospital, Toronto, Canada. The patients/participants provided their written informed consent to participate in this study.

## Author contributions

MQ and HK performed study concept and design. HK, MP, SJ, ND, MS, MR, JE, CM, MA-O, RA, and MQ performed development of methodology and writing, review, revision of the paper, provided acquisition, analysis and interpretation of data, and statistical analysis. MQ provided technical and material support. All authors read and approved the final paper.

## Funding

This research was fully funded by the Blair Foundation.

## Conflict of interest

The authors declare that the research was conducted in the absence of any commercial or financial relationships that could be construed as a potential conflict of interest.

## Publisher's note

All claims expressed in this article are solely those of the authors and do not necessarily represent those of their affiliated organizations, or those of the publisher, the editors and the reviewers. Any product that may be evaluated in this article, or claim that may be made by its manufacturer, is not guaranteed or endorsed by the publisher.
